# CFD Mechanistic Modeling of a Monolithic Chromatography System: Challenges and Opportunities

**DOI:** 10.1021/acsomega.5c11690

**Published:** 2026-01-27

**Authors:** Jamille Coelho Coimbra, Diego Martinez Prata, Raquel Nunes Fernandes, Marcio Arêdes Martins, Luis Antonio Minim

**Affiliations:** † Departament of Chemical Engineering, Federal University of Minas Gerais, Av. Presidente Antônio Carlos 6627, Belo Horizonte, Minas Gerais 31270-901, Brazil; ‡ Department of Chemistry and Petroleum Engineering, Fluminense Federal University, Rua Passo da Pátria 156, São Domingos, Niterói, Rio de Janeiro 24210-240, Brazil; § Department of Technological Chemistry, Federal Institute of Espírito Santo, Avenida Vitória, 1729, Jucutuquara, Vitória, Espírito Santo 29040-780, Brazil; ∥ Department of Agricultural Engineering, Federal University of Viçosa, Av. Peter Henry Rolfs, s/n, Viçosa, Minas Gerais 36570-900, Brazil; ⊥ Department of Food Technology, Federal University of Viçosa, Av. Peter Henry Rolfs, s/n, Viçosa, Minas Gerais 36570-900, Brazil

## Abstract

Monolithic chromatographic supports are part of the fourth generation of chromatographic separation media. A comprehensive knowledge of the transport phenomena described by mechanistic models is important for the purposes of project design for meeting regulatory requirements and optimizing production. Although geometric characteristics influence monolithic support properties, few approaches have incorporated morphology details into the models. Thus, there is a gap for new model developments in complex porous matrices, which may be alternatives to the more classic phenomenological approaches. This review differs from those previously published by demonstrating the utilization of computer-aided engineering to enhance the construction and modification of channel microstructures for the purpose of achieving more effective separation, integrating mathematical models, and constructing porous geometries. This work presents the status quo evolution of mathematical models and also presents the trends, challenges, and opportunities.

## Graphical abstract

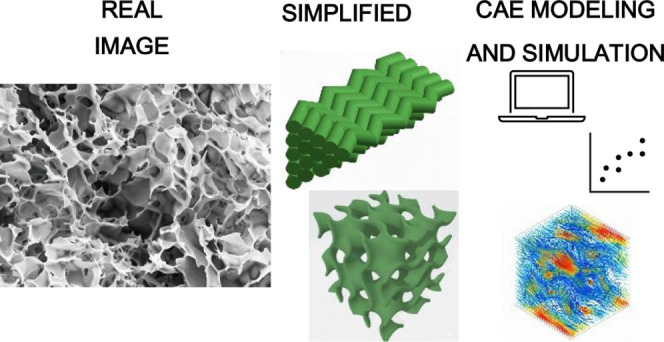

## 1 Introduction

The monolithic macroporous matrix offers new technological prospects for biological, engineering, chemical, and biomedical applications.^1–8^ Macroporous cryogels have been revolutionizing analytical chromatography by providing faster and more efficient purification.^9,10^ By dispensing the preparation steps, such as clarification, the sample can be directly purified. In parallel, new materials can be easily developed by combining reagents in the reaction mixture and incorporating binders into the matrix (functionalization) at low cost.^11,12^ At the same time, control and monitoring of process analytic technology has been increasingly encouraged, as it promotes quality and high standards in accordance with the guidelines set out by the Food and Drug Administration (FDA).^13^

Computer-aided engineering (CAE) has gained notoriety for the availability of information in less time, allowing for making better decisions, reducing time, and reprocessing costs.^14,15^ There is a great need for mathematical models that guide CAE applications, such as computational fluid dynamics (CFD) modeling, to provide intelligent prediction systems. Thus, mechanistic models deepen the process understanding, allowing for behavior prediction in the face of operational variability, through prior monitoring, to ensure optimal operating conditions and system design.^16^

With the evolution of simulation tools and computational processing power, many detail-rich approaches have been developed in robust chromatography models. However, how to portray geometric features and incorporate them into the models to precisely predict the dynamics of the chromatographic system remains a challenge.^17^ As the mathematical detailing increases, the model becomes more parametrized, with less numerical convergence, higher computational cost, and simulation time, especially in the nonlinear chromatography field.^18,19^ Therefore, different mathematical approaches with more simplified proposals have been emerging to solve this problem.

The classical mathematical models most used to represent the mass transfer phenomena of packed or monolithic chromatographic beds are the general rate model, the lumped pore diffusion model, and the equilibrium dispersion model.^20–23^ Recent developments have included details of the geometric structure to characterize and describe properties of the porous network, assessing the fluid dynamics and mass transport in the presence of intricate monolithic morphology.^17,24–26^ To date, very few models relate monolithic geometric architecture to the performance of the separation and purification process, so CFD modeling can be an alternative to the more classic phenomenological approaches. It is based on the most typical geometric features or even the actual representation of the effectively manufactured shape, whose numerical prediction can be obtained for each design component.^27,28^ Considering the efficient integration between design and simulation, there is a tendency to use the CFD model for chromatographic applications as reported by the tetrahedral skeleton,^29^ cubic lattice,^30^ capillary,^31^ gyroid,^32^ and advanced image reconstruction;^25^ however, there are still gaps to be explored.

Recent literature reviews have focused on enhancing the selectivity of composite cryogels through chemical modifications.^33^ The changes are meant to create functionalized cryogels with better properties, like hydrophobicity to separate oil and water, as well as to explore new manufacturing routes for environmental sustainability and energy production.^34^ The reviews also highlight advancements in synthesis procedures associated with structural performance, such as surface area, stability, low toxicity, tunable compositions, biocompatibility, and selectivity.^35,36^ The techniques employed in the creation of cryostructures and the various aspects that influence their characteristics.^37^ Rosseau et al. (2022) provided a critical examination of the development of novel monolithic adsorbent structures with enhanced characteristics using 3D printing or additive manufacturing. An inherent constraint of employing this methodology is the generation of intricate configurations; thus, it is recommended to primarily utilize them for the production of more straightforward geometries, such as multihole cylinders or multilobe shapes.^39^

To the best of the authors’ knowledge,^40^ in the open literature, only the review paper by Chisăliţă et al. (2025)^37^ deals with the assessment of absorbent shape for a specific process (adsorption by pressure swing) with a view to intensifying the production process based on mathematical modeling.

While the connection between the structural properties of monolithic porous materials and separation performance indicators is widely acknowledged, there is a lack of research demonstrating the utilization of computational tools to enhance the construction and modification of the channel microstructure for the purpose of achieving more effective separation.^38^ Thus, this review conducts a comprehensive analysis of CAE related to monolithic chromatographic beds that incorporate mathematical models and the fabrication of porous geometries, as it precedes the scale-up of additive manufacturing and analogous emerging technologies. Issues involving pressure drop, mass transfer resistance, permeability, residence time, and purification performance are highlighted for different geometries. In addition, the present work also highlights the challenges involved in choosing the calculation domain and using dimensional analysis to guide sizing decisions.

Therefore, based on a literature review, this paper addresses a gap in the existing literature and presents an outline of the mechanistic models utilized in monolithic chromatography beds, particularly focusing on supermacroporous cryogels. It discusses the advancements in solution techniques, the progress of simulation tools in predicting process performance, and the recent applications and verification procedures of CFD models. We will examine the status quo evolution of mathematical models as well as highlight the trends, challenges, and opportunities associated with using such models as soft sensors. Furthermore, this review aims to serve as a comprehensive guide for the effective use of monolithic chromatographic models, as information in the literature is currently quite dispersed.

## 2 Properties of Monolithic and Conventional Chromatographic Supports

Within chromatography columns, the adsorbate becomes attached to the stationary phase, as described by Keller and Giddings (2016a).^37^ Meanwhile, the mobile phase flows through the empty channels between the particles (Figure 1).

**Figure 1:**
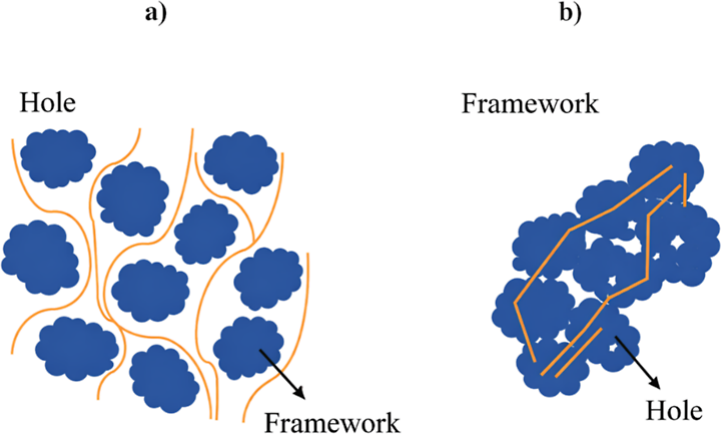
Flow path scheme in (a) a particle-packed bed and (b) a monolithic bed.

Ideally, the particle size reduction produces higher packing and narrowing of the interstitial channels. This lowers the barrier to mass transfer and the band broadening induced by the eddy diffusion. Consequently, as particle sizes are lowered, the pressure drop increases and the chromatographic performance improves.^41–43^

Considering that conventional chromatography columns operate with low flow rates due to high head loss associated with high particle packing, the scenario is opposite in cryogel columns.^44^ This occurs because the monolithic bed is composed of a single unit with interconnected macroporous void channels (up to 100 μm), where the mobile phase flows (Figure 1).^45^ Due to this particular structure, it can withstand high flow rates and maintain high permeability. Moreover, resistance to mass transfer is negligible, and the convective mechanism predominates, which is advantageous for the separation and purification performance.^44,46^

Monolithic cryogels can be produced from a polymer reactive mixture composed of hydrophilic monomers dissolved in water that react at subzero temperature (−5 to −20 °C).^47^ In this process, known as cryopolymerization, a rigid, highly porous and interconnected structure is formed due to the following steps: ice crystal formations that act as a porogenic agent, cross-linking and polymerization accompanied by melting of the ice crystals.^48,49^ A schematic representation of the cryogel manufacturing method until its application in preparative chromatography is shown in Figure 2.

**Figure 2:**
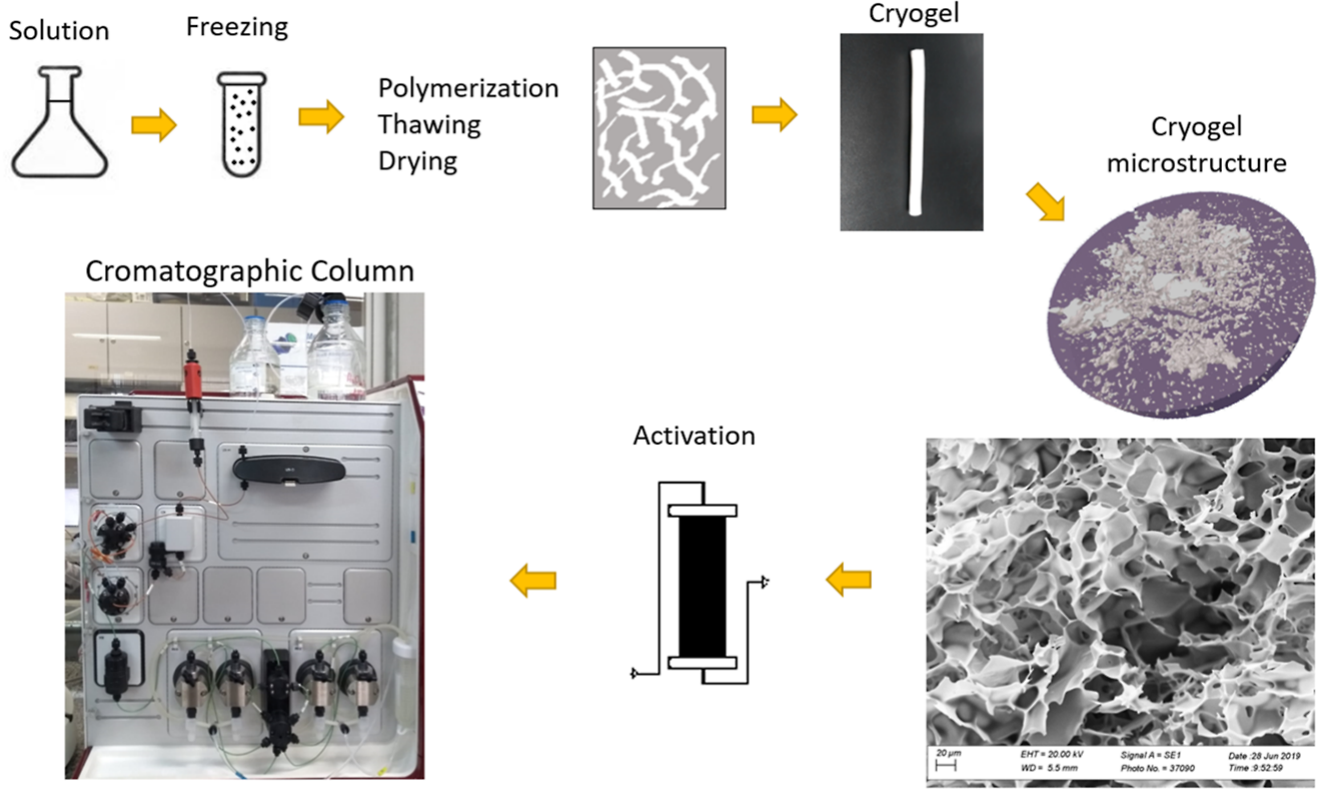
A schematic representation of the cryogel manufacturing method until its application in preparative chromatography. Adapted from ref 50.

Desirable features of monolithic columns include structural uniformity, low flow resistance, reusability, high stability, and reproducibility.^51^ Over the years, new formulation and functionality developments of cryogel supports have been extensively investigated.^52–57^ The ability to couple a variety of ligands, grafting polymeric chains to the cryogel surface^58^ or combining polymers for technological improvements, makes these adsorbents attractive and versatile. Currently, the most commercialized methacrylate-based monolithic columns are manufactured by BIA Separations under the brand names CIM and Proswift of the Dionex Corporation.^59^ Moreover, silica-based monolithic columns are preferably marketed by Merck KGaA (Chromolith) and Phenomenex (Onyx).^60^ However, many concepts such as cryogels never reached the market. Better elucidating the process conditions to scale cryogel production via computer simulation is an opportunity to increase the attractiveness of such a technology to the market.

## 3 Geometric Features of Monolithic Cryogels

Monolithic columns have an open pore structure, and the material nature is subdivided into two preferred categories: organic polymer-based or silica-based, whose preparation procedures are versatile.^61^ Among these, cryogel is a spongy polymeric adsorbent with interconnected macropores (0.1 to 10 μm) or supermacropore networks (tens to hundreds of micrometers).^62,63^ Polymer monoliths exhibit prominent macro-pores from heterogeneous polymer cross-linking^64^ with globular structure three-dimensionally adhered channels and a monomodal pore distribution. In this matrix, the soft structure of the stationary phase is considered a key factor for chromatographic performance under retention conditions.^65^ Differently, silica monoliths have a bimodal pore size distribution showing fractal bicontinuous mesoporous structure.^66,67^ Most reported geometric characteristics of monolithic matrices are described in Table 1.

**Table 1:** Geometric and Structural Characteristics of Monolithic Matrices^a^

monolithic material	surface area (m^2^/g)	pore size diameter (μm)	porosity (%)	refs
collagen cryogel	0.41	16.2–344.06	90.67	^68^
PHEMA cryogel	20.2	10–200	71.6	^69^
PHEMAH cryogel	38.6	10–100	88	^69^
cellulose composite cryogel	5 – 50	“few microns”	89 – 93	^70^
CNF cryogel	23 – 27	4–31.6	99.4	^71^
PAAm + DMAEMA cryogel		20–100	92	^72^
PAAm + AMPSA cryogel			85	^73^
PAAm + Tris cryogel		20–80	91	^74^
PAAm + Tris cryogel			87 – 95	^9^
silica-based (first generation)	320	1.8–2	>80	^75^
silica-based (2 st generation)	250	1.1–1.2	>80	^75^
Silica-based (3rd generation)		0.5–1.0	>80	^76^

a Note. PHEMAH: Poly­(hydroxyethyl methacrylate-*N*-methacryloyl-(l)-histidinemethylester), PHEMA: composition similar to PHEMAH without including MAH ligand, CNF: Cellulose nanofibrils, PAAm: poly­(acrylamide), DEMAEMA: 2-(dimethylamino)­ethyl methacrylate, AMPSA: 2-acrylamido-2-methyl-1-propanesulfonic acid, Tris: Tris­(hydroxymethyl)­aminomethane.

It can be noted that different polymeric compositions can contribute distinctly to the structural properties. The development of composite cryogels for the simultaneous adsorption of two or more species is still challenging. According to Dragan et al. (2023),^37^ coupled networks boost separation selectivity. Developing more reliable mechanistic models requires incorporating geometrical and structural details to obtain more accurate responses regarding the resistance to mass transfer, flow, and pressure profiles in the interconnected porous network. The monoliths are very useful to analyze and purify plasmids, viruses, gene therapy vehicles, and bionanoparticles, such as the recently emerged mRNA vaccines.^77,78^ They are so useful because monoliths provide a sufficient surface area and a wide enough channel diameter. CFD techniques can provide insight into the particle size on separation performance. In this sense, methodologies have been developed to improve the physical characterization of monolithic adsorbents. Table 2 illustrates experimental procedures or mathematical correlations used in the physical characterization of the cryogel.

**Table 2:** Experimental Techniques and Mathematical Models Used for Physical Cryogel Characterizations

physical characterizations	mathematical model (ref)		experimental techniques (ref)
apparent density (**ρ**)	ρ = *W*/(π×(*D*/2)^2^×*L*)	^79^	Mercury Intrusion Porosimetry^68^
porosity (**ε**)	$\varepsilon =\frac{\left(W_{2}-W_{3}-W_{s}\right)/\rho_{e}}{\left(W_{1}-W_{3}\right)/\rho_{e}}$	^79^	Mercury Intrusion Porosimetry^68,79,80^
	$$\varepsilon =\frac{V_{V}}{V_{T}}$$	^1^	
	$$\varepsilon =\frac{4\left(t_{1}-t_{0}\right)Q_{V}}{\pi D^{2}L}$$	^18^	
specific surface area (a)	$a=\frac{0.718}{d}$	^82^	Mercury Intrusion Porosimetry^68,79,80^
	$a=\frac{4}{d}$	^83,84^	BET method^69,81^
pore interconnectivity (PI)	$PI=\frac{connected\;void\;voxels}{total\;void\;voxels}$	^86^	degree of resistance directed against the flow^82,83^
	(image-processing tools)		Gravimetric method^84^
tortuosity of monoliths (τ_***i***_)	$\tau_{i}=\tau_{dmin}+\mathrm{\omega ̅}\left(d_{i-}d_{\min}\right)$	^19^	Optical methods^85^
	$\tau_{i}=\frac{{Ls}_{i}}{{Le}_{i}}$	^1^	Acoustic methods^86,87^

There is currently a growing tendency to use image processing techniques to analyze the structural architecture of monolithic materials. Hydrodynamic pore space, interconnectivity, pore size distribution, and geometric tortuosity can be obtained for comprehensive descriptions of the microstructure.^1,88–90^ Over the years, images obtained from scanning electron microscopy (SEM), confocal laser scanning microscopy, computer tomography, or microcomputer X-rays have formed a qualitative database.^91^ Nowadays, in addition to this purpose, specialized image analysis software such as ImageJ (NIH, Bethesda, Maryland, USA), AMIRA (TGS, San Diego, CA, USA), Data Viewer, CT-Vox (SkyScan, Kontich, Belgium), and VG StudioMax (Volume Graphics GmbH, Heidelberg, Germany) have been extensively used to determine geometrical parameters, composing a consistent quantitative database.^25,54,92–96^ Cryogel images obtained by scanning microCT microtomograph SkyScan1174 and by electron microscope Carl Zeiss LEO EVO 40 XVP are shown in Figure 3.

**Figure 3:**
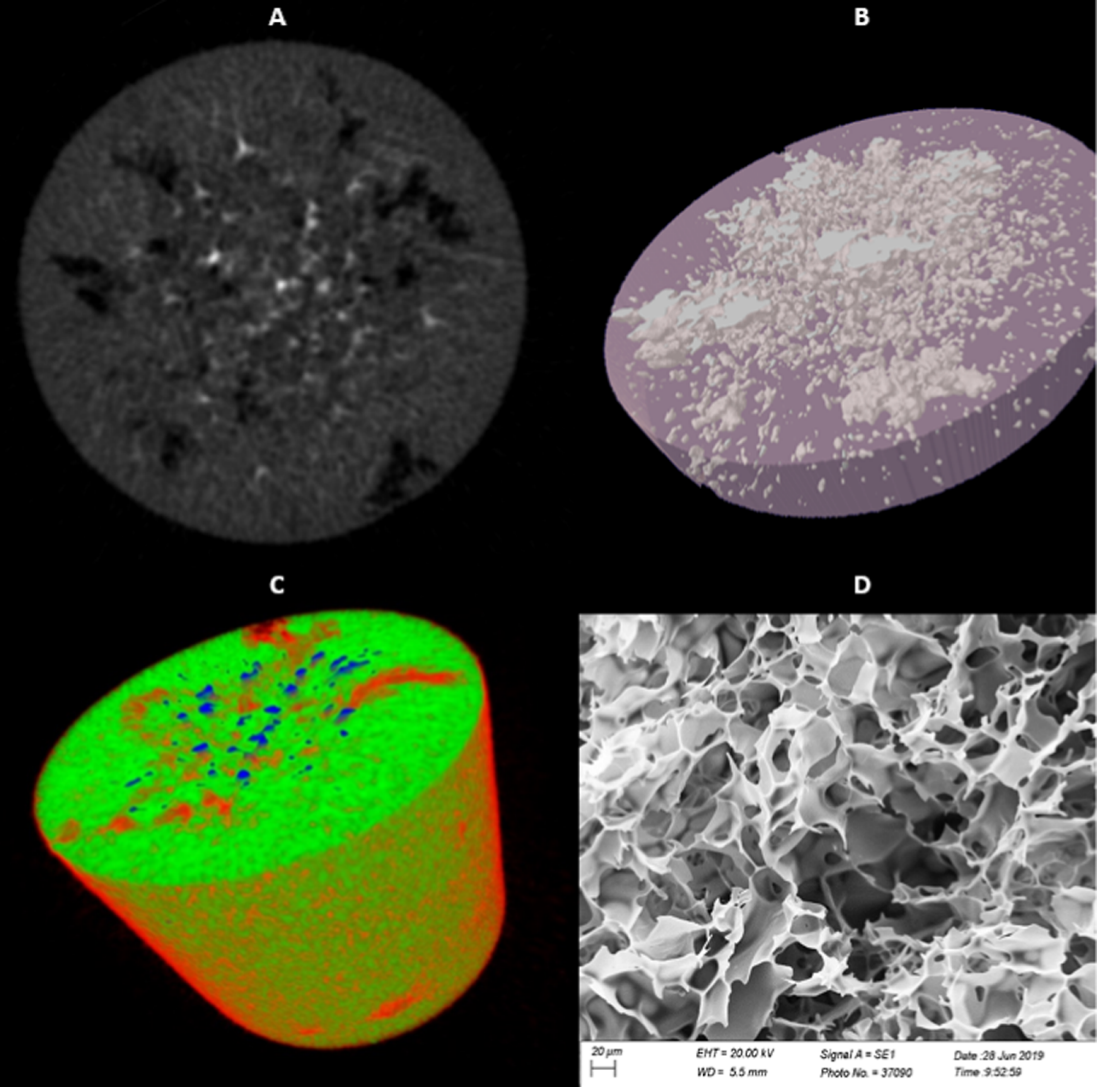
Cryogel images obtained by microCT microtomograph SkyScan1174 (a–c) and by scanning electron microscope Carl Zeiss LEO EVO 40 XVP (d).

By advancing computer system performance, imaging software has been able to capture more morphological details. Characteristics of porous media through experimental techniques such as image reconstruction and its applications for simulating different physical and chemical processes were reviewed by Xiong et al. (2016).^97^

## 4 Models Considering the Detailed Structural Properties

Material, energy, and momentum balances are considered in mechanistic models that describe the physical processes involved in chromatographic separation.^98,99^ As in other adsorption processes, chromatography exhibits spatial and temporal variations that are usually represented by deterministic, mass-balance-based macroscopic models.^100^ These models require either the assumption of rapid equilibrium between the mobile and stationary phases or the explicit consideration of adsorption/desorption kinetics and mass transfer resistances, as shown in Figure 4 and Table 3.

**Figure 4:**
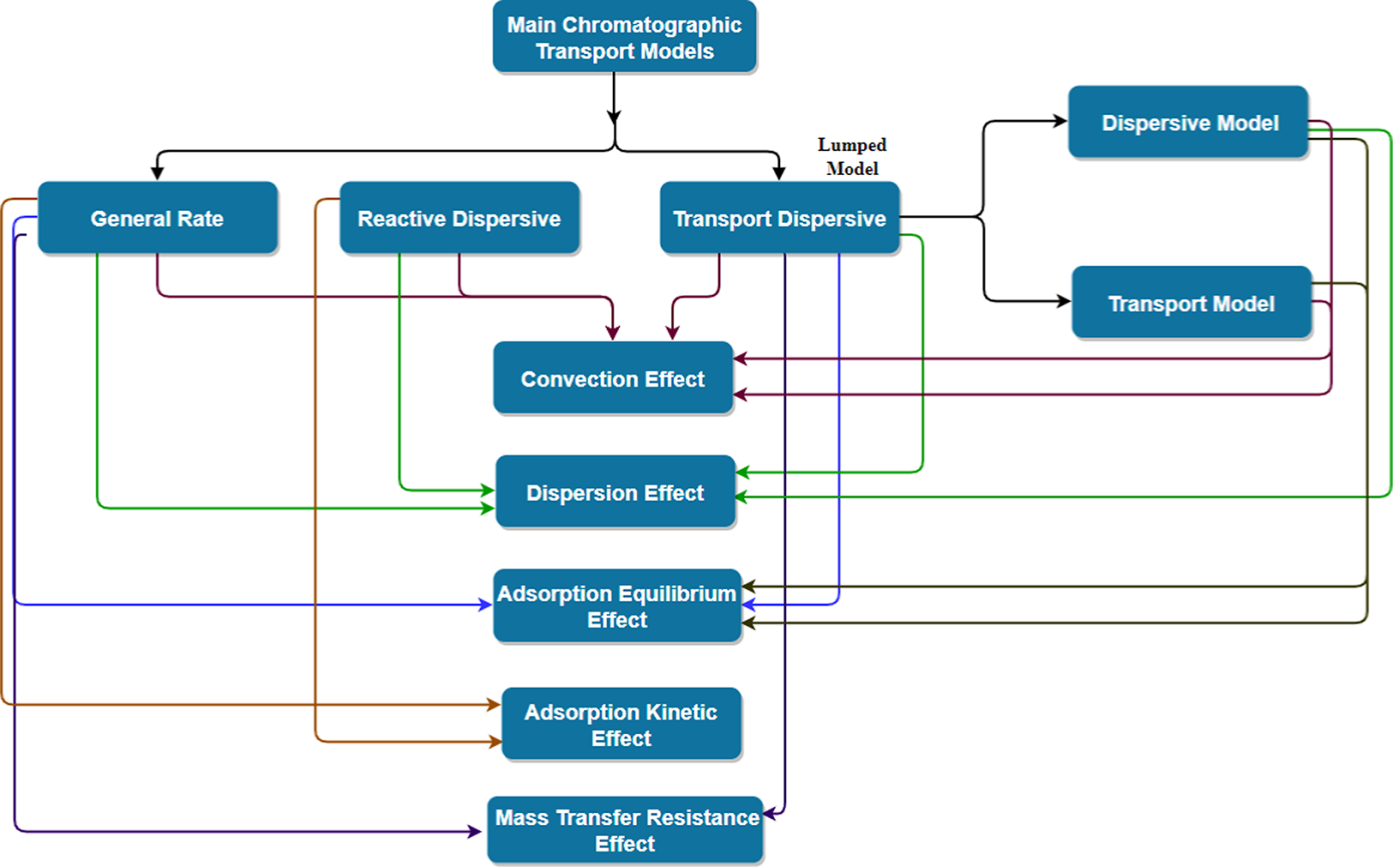
Classical mass transport models for liquid chromatography.

**Table 3:** Mathematical Formulation of Classical Mass Transport Models (Figure 4)­^a^

phenomenon	equation term(s)	symbols
convection	$=u\frac{\partial C_{i}}{\partial z}$	*u*: linear flow velocity
		*C*_*i*_: solute concentration in the mobile phase *z*: Axial coordinate
dispersion	$=D_{A,i}\frac{\partial^{2}C_{i}}{{\partial z}^{2}}$	*D*_*A*,*i*_: dispersion coefficient
accumulation	$=\frac{\partial C_{i}}{\partial t}$	*t*: retention time of solute
adsorption equilibrium	$q_{i}=\frac{q_{m,i}KC_{i}}{1+KC_{i}}$ (Langmuir)	*q*_*i*_: solute concentration in the stationary phase
	*q*_*i*_ = *KC*_*i*_^1/*n*^ (Freundlich)	*q*_*m*,*i*_: maximum adsorption capacity
	$q_{i}=\frac{q_{m,i}K{C_{i}}^{1/n}}{1+K{C_{i}}^{1/n}}$ (Sips)	*K*: ratio of adsorption to desorption rate constants $x:\frac{C_{i}}{C_{s}}$
	$q_{i}=\frac{q_{m,i}KC_{i}}{{\left[1+{\left(KC_{i}\right)}^{m}\right]}^{1/m}}$ (Tóth)	*C*_s_: maximum solubility *n*,*m*,*c*: constants
	$q_{i}=\frac{q_{m,i}cx}{\left(1-x\right)\left(1-x+cx\right)}$ (Bet 2 parameters)	
	$q_{i}=\frac{q_{m,i}cx}{\left(1-x\right)}\left[\frac{1-\left(n+1\right)x^{n}+nx^{n+1}}{1+\left(c-1\right)x-cx^{n+1}}\right]$ (Bet 3 parameters)	
adsorption kinetic	$\frac{\partial q_{i}}{\partial t}=k_{a,i}\left(q_{m,i}-q_{i}\right)C_{i}-k_{d,i}q_{i}$	*k*_*a*,*i*_: adsorption rate constant
		*k*_*d*,*i*_: desorption rate constant
mass transfer resistance	$\frac{\partial q_{i}}{\partial t}=k_{m}\left[q_{i}^{*}\left(C\right)-q_{i}\right]$	*k*_*m*_: mass transfer coefficient
		*q*_i_^*^(*C*): isotherm stationary phase concentration in equilibrium with local mobile phase concentration

a The dispersive and transport models account for mass transport resistance indirectly, and the kinetic parameters while properly used can lump various kinetic contributions to band broadening^101^.

Classical models (Figure 4) applied to fixed bed chromatography have been extensively used to describe the mass transfer mechanisms. Typical steps involved in model development for preparative separations are summarized in Figure 5.

**Figure 5:**
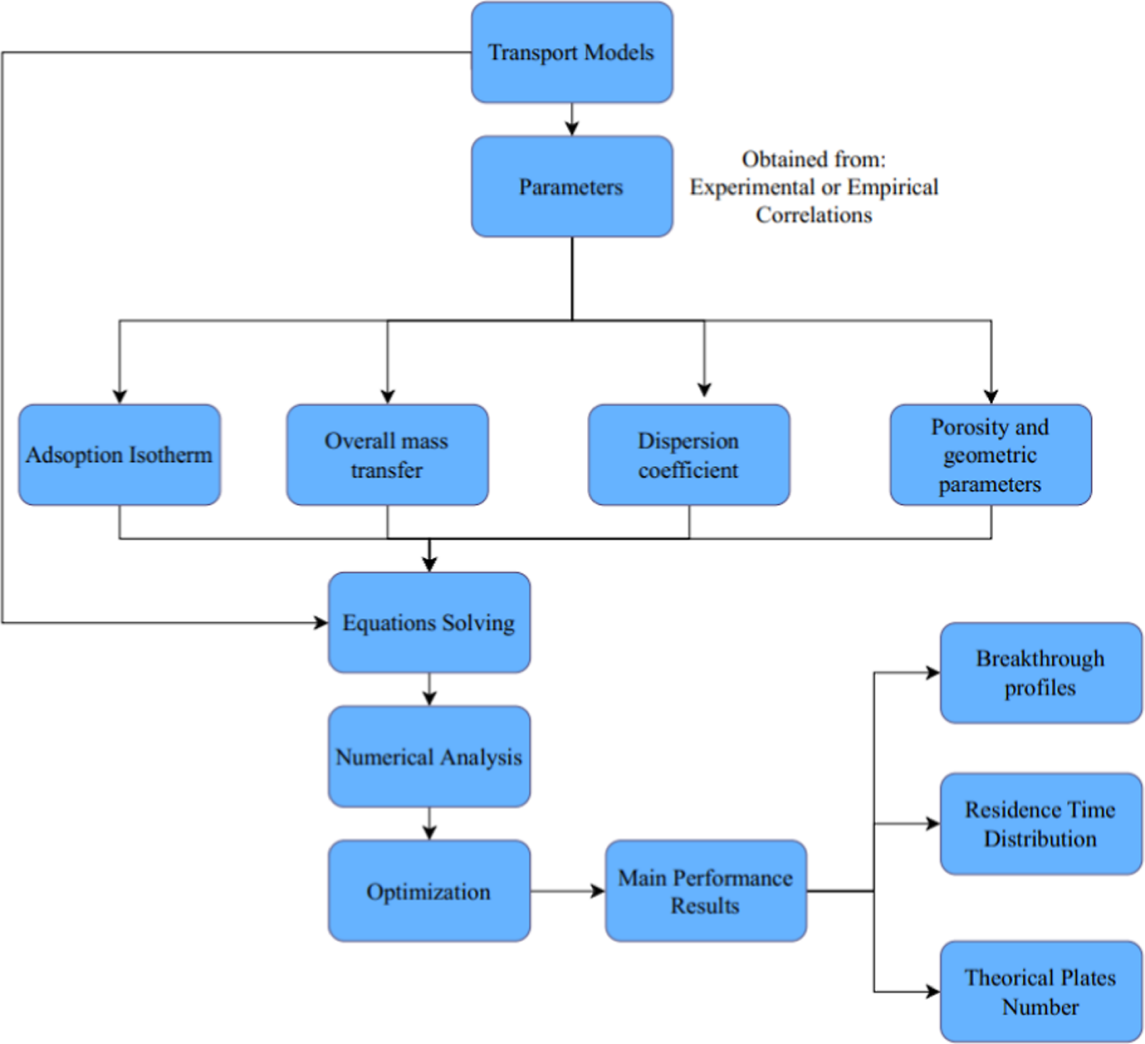
Representation of steps frequently used during the modeling development of chromatography columns.

A comprehensive review of mechanistic transport and adsorption models in liquid chromatography was provided by Shekhawat and Rathore (2019).^37^ Although these models are most commonly applied to packed beds, they are equally applicable to monolithic formats. In this context, the impact of morphology on flow behavior, residence time distribution, and solute dispersion is addressed in this work.

Because real porous media exhibit a highly complex and chaotic structure, an exact geometric reproduction is impractical. Therefore, simplified models capable of reproducing key geometric properties—such as surface area, porosity, pore size distribution, tortuosity, and interconnectivity—within acceptable error margins are required. Accordingly, this section reviews mechanistic models that explicitly incorporate the morphological details of monolithic beds.

### 4.1 Macroscopic Approaches and Simplified Network Models for Structural Properties

A cubic lattice model was the pioneer in portraying an interconnected monolithic bed. It was constructed by Meyers and Liapis (1999)^37^ under different retention conditions. With more connectivity between monoliths, interstitial velocity and diffusivity into pores were significantly increased. The interstitial flow velocity was much larger than the molecular diffusion velocity, according to the authors, validating the dominant convective flow theory in monolithic beds, which favors mass transfer. Highly interconnected monolithic matrices with adequate mesopore distribution were defined to determine better separation conditions. CFD models are efficient in predicting flow patterns and dominant mechanisms (convection, diffusion, and kinetics) in different pore structures, due to the possibility of retrieving values for variables such as pressure and velocity at the local level, helping to identify the optimal conditions of the elution process.

CFD is very useful to predict the pressure drop and flow profiles within a packed bed. The big challenge about pressure drop prediction is the definition of a universal characteristic dimension that represents the monolithic structure.^25,102^ Once the geometry is known, it is possible to acquire an insight into a flow regime as well as stationary and mobile phases properties.^103^ Furthermore, information about the adsorption process on the matrix can be gained. Based on an experimental structure acquired using serial block face scanning electron microscopy (SBEM), Jungreuthmayer et al. (2015)^37^ calculated flow profile and the pressure drop. Then, CFD was used to predict better performing structures with respect to lower pressure drop. In the field of catalysis, a lot of monolith structures have been investigated to simulate pressure drop and flow profiles.^104,105^ This knowledge is also useful to understand chromatography columns.^106^ Mihelič et al. (2005)^37^ hypothesized that the structure of the monolith flow path has a substantial impact on pressure drop.

More closely analyzing the hydrodynamics of silica monoliths, Vervoort et al. (2003)^37^ proposed a geometric simplification model based on the tetrahedral skeleton structure (a network of interconnected cylinders) which allowed for predicting the variation of flow resistance according to structural parameters of the monolith thickness and bed porosity. Subsequently, Vervoort et al. (2004)^108^ modified the porous network topology by introducing varying distances between channels, simulating irregular monoliths. Pore uniformity influenced the chromatographic separation efficiency much more than pore size in the monolithic matrix. Complementing the previous study, Vervoort et al. (2005) included the interconnectivity structure analysis to evaluate the correlation between structural heterogeneity and flow resistance.^135^ For this purpose, two skeleton models were developed: the homogeneous model with uniform diameter and the heterogeneous model with different diameters and a superior number of interconnected channels. By increasing the heterogeneity of the fluid path, a gradual reduction of the flow resistance occurred. However, the impact of incorporating heterogeneity through geometric modification. In terms of bioseparation, the creation of preferential pathways due to increases in the flow heterogeneity shortens the residence time of adsorbate molecules, which can be disadvantageous and should be evaluated during kinetic and mass transfer analysis of monolithic beds. Recently, Jacquot et al. (2023)^109^ developed a CFD model of a packed spherical catalyst bed and a monolithic bed. The first geometry demonstrated a heterogeneous flow (swirly flow and displaced RTD). On the other hand, the second geometry demonstrated a homogeneous flow pattern (creeping flow and uniform RTD) with a shorter residence time and, thus, a higher conversion rate.^109^

Residence time distribution (RTD) analysis provides valuable insight into flow patterns, mixing efficiency, and heterogeneities. In a classical approach, RTD is determined by monitoring the tracer concentration at the exit of the chromatographic column or by mathematical modeling by solving flow and mass balance equations for this tracer.^110^ On the other hand, spatial and temporal RTD solutions within a flow field can be obtained through CFD according to the so-called Smart RTD (SRTD) method proposed by Simcik et al. (2012)^111^ to determine the instantaneous age of the fluid particle at each position in space.^112^ This technique is relevant for predicting RTD and the axial dispersion coefficient (calculated from the RTD of the eluent) in heterogeneous flow, as often occurs in cryogels. In these matrices, axial dispersion (Da) is derived from the contribution of molecular diffusion and eddy dispersion (the result of a nonuniform velocity in the axial direction due to irregularity of the monoliths or interconnections). Thus, knowing the SRTD provides insights into the mixing, flow maldistributions, and adsorption efficiency (dynamic capacity) at each point in the porous structure.^111^

In this context, Brenner’s dispersion theory is a theoretical approach that refines the description of the dispersion of a solute in a moving fluid, taking into account the interactions between advective transport (driven by flow) and diffusive transport (driven by concentration gradients).^113^

While the classic Taylor-Aris model describes dispersion in pipes assuming a quasi-stationary solution with balanced transverse diffusion, Brenner generalized this concept to include (i) broader conditions of geometry and flow regimes, (ii) corrections for flows that are not fully developed, and (iii) boundary effects and temporal variations in the velocity field. Brenner proposed that, under certain conditions, effective dispersion can be treated as additional diffusion, and he mathematically formalized how to calculate this dispersion coefficient based on flow and medium properties.^113^ The Brenner’s dispersion formalism in chromatographic media is presented by Venditti et al.(2023).^114^

Minimizing axial dispersion and backmixing is essential for improving separation efficiency and is strongly linked to the porous morphology. In this regard, the CFD supports the prototyping of monolithic columns by integrating structure generation, RTD analysis, and dispersion coefficient estimation. The design of adsorbents can be increasingly optimized by evaluating these pillars.^25,115^

Sometimes, the pore structure cannot be morphologically described, and the macroscopic approach is a tool to investigate fluid dynamic properties at the global level. Thus, the fluid flow is theoretically investigated in a sample elementary volume by employing Darcy, Brinkman-Darcy, and Forchheimer–Darcy equations based on macroscopic properties that constitute parameters of the model.^116^ The silica matrix presents an ordered, uninterrupted sequence of long channels. The polymeric stationary phase, on the other hand, generally presents micro or macro globules (e.g., cryogels) between the monoliths.^65^ The backmixing effect is expected to be less pronounced on silica supports compared to polymeric supports with the same pore size due to the higher homogeneity of this structure. Macroscopy medium is more suitable to describe the first case. However, a macroporous network with open porosity is verified in polymeric cryogels, which favors the advection as compared to diffusion, reducing the backmixing, and the macro approach can be applicable, as reported by Coimbra et al. (2022).^50^ In order to investigate if the macroapproch is suitable to portray the chromatographic model, it is recommended to plot the RTD and breakthrough curves. Symmetrical RTD peaks and step-shaped breakthrough profiles are good indications that the geometry is homogeneous, without formation of preferential paths, and exhibits low axial dispersion.

### 4.2 Mathematical Models Applied to Pore Scale Local Analysis

Otherwise, there is also the pore-scale approach, in which real porous structures are directly modeled using mass and momentum conservation laws, requiring high computational resources.^117–121^ This strategy applies very well to monolithic supports that exhibit structural heterogeneity and high morphological complexity (varied pore sizes, tortuous and interconnected monoliths, etc.). By explicitly considering geometric details, it becomes possible to track fluid pathways within the porous matrix and evaluate transport phenomena at the channel level.

Numerically, porous-based models can be solved by finite element methods, finite differences, and finite volumes with complex interface discretization challenges. The Lattice Boltzmann method, widely used in porous media, overcomes the problem of the discretization step by representing particle movement through microscopic kinetic equations that represent particle distribution functions. Massive parallel computing is required to provide detailed information at the microscale level.^120,122^ The Lattice Boltzmann method from reconstructed images was used in a porous domain of 60 μm × 60 μm × 12 μm,^123,124^ focused on evaluating the length scales of eddy dispersion in 100 μm i.d. capillary silica monoliths,^125^ chromatographic band broadening,^126^ and transient dispersion behavior.^127^

Regarding the pore scale approach, the strategy of analyzing subdomains proposed by Pawlowski et al. (2018)^25^ reduced the aspect ratio and the computational cost. The CFD model included scalar transport and Navier–Stokes equations to analyze flow patterns, tortuosity, and residence time distribution. For this purpose, a monolithic silica column was reconstructed using X-ray tomography data, and two arbitrary subvolumes (=1.21 × 10–3 mm3 and 2.85 × 10–3 mm3) were chosen to perform CFD simulations. According to Koku et al. (2012),^128^ it is feasible to use direct image-based techniques for chromatographic modeling by capturing the high resolution microstructure in a polymeric monolith bed. However, the rigorous and explicit geometric approach presents challenges regarding the selection of representative sample volumes to depict physical phenomena. Silica monoliths are similar to a network of through pores connecting small pores with active adsorption centers, and the primary solute transport is due to diffusion. In contrast, the organic polymer monoliths contain a very low proportion of tiny pores, and the solutes are mainly retained by a convection process.

Including all the morphological details of the porous media geometry in CFD simulations often becomes unfeasible due to discretization limitations (proper element shape, need for high mesh refinement, etc.). The strategy proposed by Pawlowski et al. (2018)^25^ uses subdomains that should represent the entire structure very well. Another widely used methodology analyzes the entire porous matrix at the pore level from simplifications of the geometry, as described below.

Based on the idea of a cylindrical capillary bundle, but exploring new geometric aspects (curvature and tortuosity), an approach proposed by Coimbra et al. (2020)^37^ allowed for better understanding of the transfer phenomena in cryogel support. A simplified CFD model was created to assess the impact of channel type and tortuosity on the fluid dynamics and purifying conditions in cryogel columns. An assessment was conducted to determine the impact of the channel structure on the scalar transport phenomena in four different monolith shapes with varying levels of tortuosity. Therefore, the construction featuring convoluted cylindrical channels arranged in a triangle pattern and a tortuosity of 1.12 exhibited a more accurate alignment with the experimental data. The aforementioned models are included in the CFD category, whose geometries are portrayed as part of the mathematical model. In contrast, the capillary model described by Persson et al. (2004) portrayed polymeric cryogels based on transport through aligned, noninterconnected, and convoluted open tubes. In Yun et al.^130^ and Yun et al.(2011),^18^ additional structural pieces of information were incorporated into the capillary model. This included several estimations for the quantity of capillaries, pore dimensions, and tortuosity ranges. Governing equations were solved progressively and repeatedly for numerous capillaries (>2000), resulting in a considerable processing cost since they were applied to an overparameterized model.

Adding more complexity to the capillary-based geometry, supermacroporous cryogels were represented for the first time using a triply periodic minimal surface (TPMS) structure known as Gyroid.^32^ Similar to cryogel, the Gyroid is a highly porous structure with a preferably axial flow pattern, three-dimensional tortuosity with interconnections, and channel rotations. The adsorption process was impacted by changes in velocity profiles, and these profiles were affected by the geometry. Moreover, the geometry of the monolithic medium produced acceleration profiles, fluid deceleration, vorticity, and other phenomena that can promote different responses to the mass transfer process. This approach was innovative in portraying similar morphological characteristics between Gyroid and cryogel via CFD. Although the first geometry does not present all of the details of the second, the geometric detailing was sufficient to capture the essential features of mass transfer and fluid flow as demonstrated by the validation results. Geometric representations of the tortuous capillary model, capillary bundle, and gyroid are shown in Figure 6.

**Figure 6:**
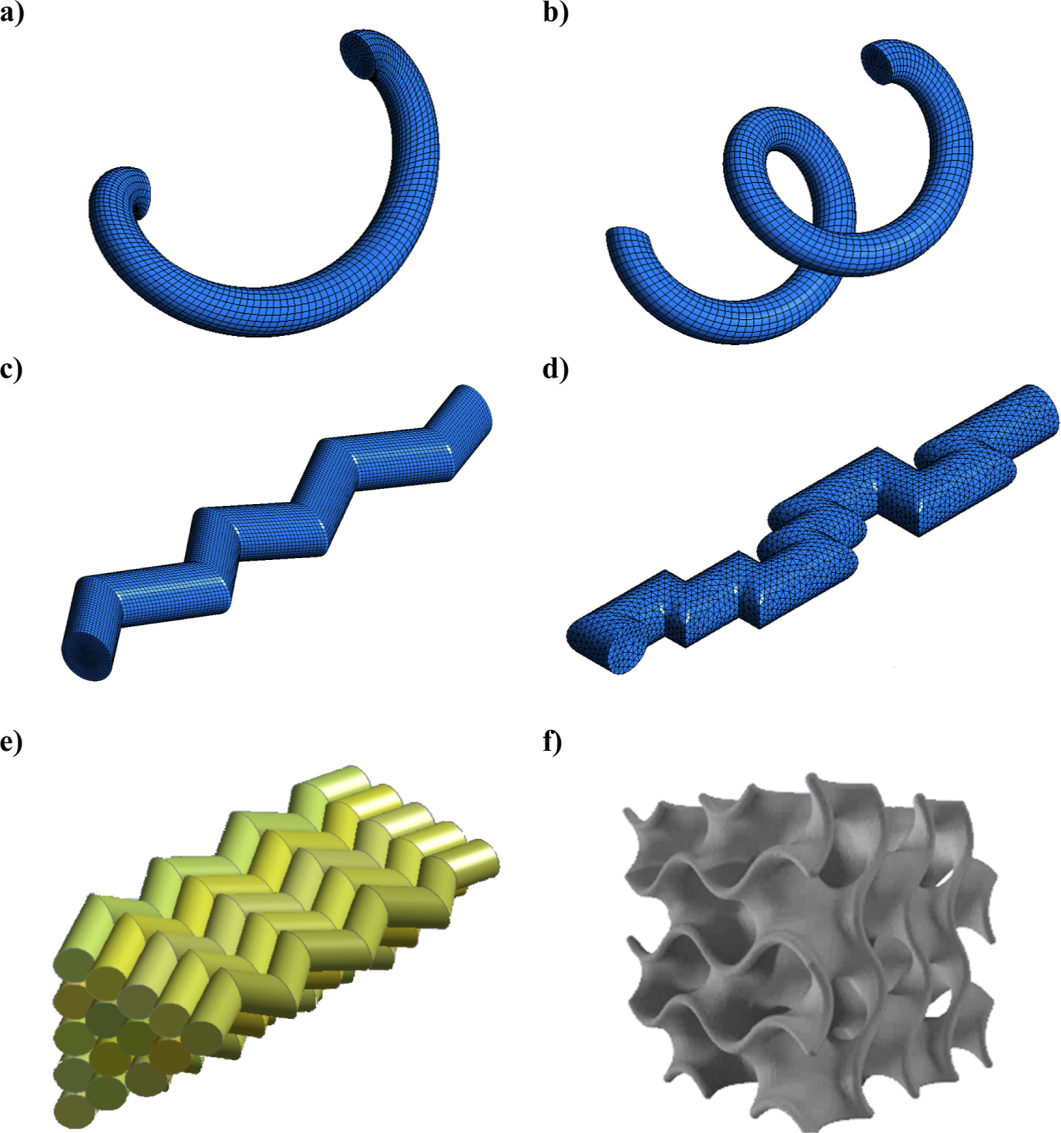
Geometric representations of the tortuous capillary model (a–d), capillary bundle (e), and gyroid (f) reported previously in refs 31 and^32^ . Adapted from ref 31 and ref 32.

Local CFD analysis of transport phenomena is promising due to its ability to evaluate morphology within specific subdomains, addressing aspect ratio limitations arising from distinct pore and channel length scales. CFD models enable transport analysis by using either reconstructed geometries or simplified representations. This last strategy was adopted by Coimbra et al. (2021)^32^ to represent polymer cryogel using gyroid structures, which describe an interconnected pore network with periodic channels and three-dimensional tortuosity. This approach reduced parametrization and computational cost by analyzing a limited number of channels. It is possible to substantially simplify the geometry of the monolithic porous network at the channel level, as proposed by Coimbra et al. (2020)^31^ since the same concentration distribution in an individual capillary or in an ensemble of tortuous capillaries was produced.

In cryogel chromatographic supports, the mathematical formulation of mass and momentum transport mechanisms commonly used is represented in Table 4 and it is also applicable to other monolithic media.^18,31,32,50,129,130^ The transport equation is described in dimensionless format, including convection (Péclet term), dispersion (Péclet term), accumulation (time derivative terms), mass transfer resistance (Stanton term), and adsorption equilibrium (isotherm term). The eqs 1 and 2 are continuity and Navier–Stokes dimensionless, respectively, and eqs 3–14 (Table 4) are derived from the governing equations presented in Table 3.

**Table 4:** Overview of Mathematical Modeling of Monolithic Supports^a^

mathematical modeling	N°
1 ∇·u=0	(1)
2 $\rho \frac{Du}{Dt}=\rho g-\nabla p+\mu \nabla^{2}u+\frac{\mu u}{k_{w}}$	(2)
3 $C_{D}=C/C_{o}$	(3)
4 $t_{D}=t_{u}/\varphi L$	(4)
5 $Pe=Lu/D_{ax\varphi }$	(5)
6 $q_{D}=q/C_{o}$	(6)
7 $\frac{DC_{D}}{Dt_{D}}=\frac{1}{Pe}\nabla^{2}C_{D}-\frac{\partial q_{D}}{\partial t_{D}}$	(7)
8 $St={ak}_{f\varphi }L/u$	(8)
9 $C_{D}^{*}=C^{*}/C_{o}$	(9)
10 $\frac{\partial q_{D}}{\partial t_{D}}=St\left(C_{D}-C_{D}^{*}\right)$	(10)
11 Re=ρud/φμ	(11)
12 $Sc=\mu /\rho D_{AB}$	(12)
13 $k_{f}=\gamma \frac{D_{AB}}{d}{\left(ReSc\frac{d}{\tau L}\right)}^{1/3}$	(13)
14 $q_{D}=k_{ad}C\backslash D^{*}$	(14)

a Mathematical modeling proposed by Coimbra et al^32,50^ .

New geometry approaches for portraying monolithic porous material have been investigated.^97,109,131–133^ However, few applications are found for cryogels due to their sponge-like and highly interconnected structure that challenges their representation.^134^ CFD models of monolithic chromatographic beds, especially cryogels, still have some gaps to be explored, such as the link between flow properties and separations, geometric representation, definition of the aspect ratio, and axial dispersion models that take into account geometric complexities, among others.

Recently, six triple periodic minimal surface (TPMS) models—gyroid, diamond, primitive, and sheet forms for each—were examined by Adrover et al. (2024) to replicate a monolithic silica adsorbent. All models demonstrated strong predictive capability for the effective diffusivity parameter, while the performance of the sheet geometry was slightly inferior. The study was confined to examining solely diffusive characteristics, excluding the convective effects related to axial dispersion, for instance. According to the authors, the actual challenge is to obtain more experimental data needed to obtain a more accurate representation of the porous medium, especially for the geometric parameters of the specific surface area, pore connectivity, mean curvature, and external porosity.

To address limitations in experimental geometric characterization, Zhou et al. (2024) used a chord length distribution (CLD) study obtained from stereological analysis on the reconstructed monolithic column of two poly­(styrene-*co*-divinylbenzene). A quantitative assessment of the 3D pore space and microglobule clustering network was conducted to understand the chromatographic transport phenomena. The authors quantify the intersection of line segments with the pore and substrate phases in the image, applying a modified Bresenham technique through subpixel path intersection as a versatile representation of pore size distribution. Moreover, the geometric tortuosity was computed by the ratio of Euclidean distance between 2 planes and the total of geodesic distances. Both evaluations were carried out in a Python script, demonstrating that this approach can serve to extract morphological properties a priori to CFD implementation. Despite the tendency to employ reconstructed geometry to derive morphological and fluid dynamic features, the homogenization technique remains extensively utilized.^17,50,136,137^ This methodology involves substituting the micromeso domains characterized by repetitive geometric features with a homogeneous domain that reflects the average response of the composite at a macroscopic scale.

In turn, a comprehensive investigation of eight TPMS geometries enhanced previous studies regarding the kinetic and adsorptive efficacy of monolithic chromatographic columns.^138^ The authors examined three support-based triply periodic minimal surfaces (TPMS): schoen gyroid (G), schwarz diamond (D), and schwarz primitive (P), as well as five sheet-based TPMS: two sheet gyroids (SGa and SGb), two sheet diamonds (SDa and SDb), and one sheet primitive (SPb), all possessing an internal porosity of 0.4. Except for geometries affected by preferential pathway formation, the best-performing geometries, namely, SDa and SGb, are associated with lower values of effective diameter and thus lower values of permeability. When kinetic performance factors are compared, the best-performing geometries are SPb, D, and SGb, which have lower tortuosity and a better coefficient of uniformity of the axial velocity field.

Optimizing morphological and operational characteristics is a precursor to building a design with better performance. Erfani et al. (2023)^138^ proposed a topology optimization for monolithic catalysts in a packed-bed reactor with an endothermic reaction, utilizing a CFD module in COMSOL Multiphysics. The design that achieved the maximum performance (222% more conversion and 19% less pressure drop) comprises fin-like elements, channels close to the heat source, and curves with holes in the surfaces. Moreover, gas diffusion in the monolithic support increased and, consequently, the mass transfer. Another approach to optimizing the performance in a monolithic structure was proposed by Huo et al. (2022).^139^ The authors developed an innovative method to create monolithic catalysts with biomimetic spiral porous structures of nautilus and intricate geometries, achieved by integrating 3D printing with CFD tool design.^140^ Exhibiting enhanced specific surface area and adjustable porosity, 3D-printed scaffolds were produced by utilizing biomimetic technology and the direct ink writing (DIW) approach in a single step process. The influence of different channel offset angles, axial channels, and radial channels on the 3D printed monolithic support during the catalytic reaction of ethylene oxidation to ethylene glycol was analyzed alongside CFD fluid simulation.

The structure–performance relationship can serve the CFD platform and auxiliary computational tools to predict a wide spectrum of morphological descriptors to better the speed and accuracy of chromatography analysis, which would enable a major technological advance in the direction of “Predict, Design, and Produce.”

## 5 Model Validation

The quantification of uncertainties and rigorous validation of CFD models are indispensable in monolithic chromatography to ensure the predictability of mass transfer and hydrodynamics within complex geometries. These processes facilitate the translation of inherent additive manufacturing variability into robust design parameters, thereby ensuring high precision in component separation and adsorption kinetics. Accordingly, a comprehensive range of studies documenting CFD model validation and the impact of parameter uncertainties in the context of flow and mass transfer in monolithic supports are presented herein.

The first validation front in building the most suitable monolithic geometry involves pressure drop comparison of the obtained flow resistance values with experimental data, and indirectly obtaining the permeability.^29,107,141^ Recently, Reinao et al. (2025)^141^ validated a dual-cell monolith model using Hagen–Poiseuille benchmarking and grid independence tests. By incorporating physical measurements to account for 3D-printing (DLP) inaccuracies, the study addressed permeability deviations of 9%–26%, though it focuses strictly on nonreactive hydrodynamic pressure drop.

The second validation front addresses adsorptive monoliths by evaluating the mass transfer kinetics and breakthrough behavior. The model’s reliability can be established by correlating CFD predictions with experimental breakthrough curves, focusing on breakthrough time and sigmoidal (S-shape) profile.^31,32,50,142,143^ This validation is typically quantified using statistical metrics such as the coefficient of determination (*R* ^2^), the mean absolute relative error, or the relative error.^31,32,50,142,144^

Lastly, the third validation stage involves analyzing the residence time distribution (RTD) to characterize the flow patterns within the structure. Although the axial dispersion coefficient is typically derived from RTD curves, experimental validation for these monolithic frameworks remains underrepresented in the literature, as seen in the limited studies by Pawlowski et al. (2018)^25^ and Heibel et al. (2005).^145^ Pawlowski et al. (2018)^25^ validated the residence time distribution (RTD) in monolithic porous columns reconstructed from X-ray tomography data to evaluate flow patterns and separation efficiency indicators. Although quantitative metrics were not explicitly reported, the study provided a qualitative benchmark by comparing experimental visual data against numerical model results.

Complementing the validation process, sensitivity analysis serves as a critical tool to enhance the model’s robustness. This analysis enables the identification of key governing parameters, supports model calibration, and reduces uncertainty in CFD-based predictions for adsorptive monolithic systems. For instance, to mitigate parameter uncertainties, Zhu et al. (2025)^146^ performed sensitivity analyses on effective diffusivity and isotherm models, while manufacturing tolerances were addressed by calibrating the simulation geometry through scanning electron microscopy (SEM) characterization. Furthermore, Coimbra et al. (2021)^32^ demonstrated that flow velocity and axial dispersion coefficients are primary drivers of flow heterogeneity within the monolithic framework. Such conclusions were substantiated through the experimental validation of residence time distribution (RTD) profiles complemented by a systematic sensitivity analysis assessing the influence of these parameters on the overall RTD response.

## 6 Scaling Analysis

Scaling analysis is defined as a systematic mathematical technique based on the Buckingham π Theorem and dynamic similarity principles. It is employed to identify the dominant physical mechanisms within complex systems by evaluating the order of magnitude of individual terms in the governing equations.^147^ As a tool to verify prevailing physical phenomena, it typically follows the nondimensionalization of the mathematical model.^148^ This compact formulation enables the determination of key variables that most impact the system behavior, facilitating strategic simplifications that reduce numerical complexity while maintaining model integrity.^149–151^ A comprehensive review of scaling analysis as a verification procedure is recommended for further reading in Grace and Taghipour (2004).^152^

The implementation of scaling analysis within this framework addresses four strategic imperatives that are essential for monolithic chromatography modeling.

i) Model simplification and computational efficiency: Identifying the main mass and momentum transfer mechanisms allows for modeling simplifications and numerical complexity reduction. By nondimensionalizing the mathematical model, scaling analysis reduces complex transport phenomena into universal dimensionless groups—such as the Péclet and Stanton numbers—which dictate the system’s regime and govern the strategic simplification of the numerical domain.^32^ This strategy is advantageous, especially for the modeling of monolithic chromatographic beds due to the several parameters required to describe it. There is a tendency to reduce the model parametrization as proposed by Liu et al. (2019)^17^ and Coimbra et al. (2020)^31^ in order to decrease the computational cost and make predictions feasible for different applications. Scaling analysis can be used to capture essential system characteristics with fewer parameters.
ii) Identifying dominant transport mechanisms: Scaling analysis acts as a diagnostic tool to isolate governing transport forces, providing the physical justification for model simplifications. By identifying dominant regimes—such as the transition from diffusion to advection—this methodology yields a minimally parametric model. For instance, the scaling analysis reported by Coimbra et al. (2021)^32^ revealed the predominance of the advection, represented by the Péclet number, and low resistance to mass transfer (Stanton number) applied to chromatographic separation on cryogel supports. In this sense, the adsorption kinetics could be greatly simplified due to its small order of magnitude in the case studied.
iii) Guiding sensitivity and design: Besides providing understandable descriptions, the scaling analysis can guide the sensitivity analysis by choosing the parameters or input variables that most affect the system’s outputs.^153,154^ Optimal values and uncertain operating conditions can be more easily determined. Additionally, new scaling parameters can be introduced as design criteria for efficient adsorption beds, as reported by Mohammed et al. (2018),^155^ supporting new discoveries applied to chromatographic modeling or even justifying approximations of mathematical formulation. According to Van der Sman (2008),^156^ the scaling analysis demonstrated to be a consistent criterion for determining significant model parameters under different heat and mass transfer regimes in packed beds.
iv) Dynamic similarity and reduced control volumes: Especially in monolithic media, the domain’s length scales (computational) cannot have the same dimensions as porous channels, and the similarity analysis serves as a guideline for a representative equivalent model configuration on a distinct time-space scale. Berger et al. (2021),^157^ Coimbra et al. (2020),^31^ Coimbra et al. (2021),^32^ and Ercan^158^ have applied similarity analysis in order to compare transport phenomena between benchmark and prototype physical systems.

The analysis of transport phenomena within interconnected porous networks is inherently constrained by complex morphological attributes such as high-aspect-ratio monolithic channels and irregular void distributions. While geometric reconstruction techniques utilizing high-resolution imaging offer structural fidelity, their implementation is often hindered by prohibitive computational costs.^25^ Consequently, scaling analysis emerges as a robust alternative for capturing fundamental transport characteristics within reduced control volumes, particularly in CFD-based frameworks.^32^ By requiring significantly lower computational overhead, these methodologies facilitate the development of parsimonious mechanistic models, advancing the state-of-the-art in chromatographic process design.^17,50^

## 7 Numerical Methods

Different numerical methods have been applied to simulate chromatographic elution by solving transport equations. Due to the inherently nonlinear nature of chromatographic models, analytical solutions are generally not feasible, making numerical approaches essential. Commonly employed techniques include orthogonal collocation,^159,160^ finite element (FE),^161,162^ moving finite element method (MFEM),^163,164^ finite volume (FV),^165,166^ finite difference (FD),^167,168^ and even coupled methods,^169,170^ which can also be applied to the simplified models presented in Figure 4 and Table 3.

In the numerical solution procedures, the governing partial differential equations (PDEs) are discretized in space using a computational mesh and in time through temporal subdivision. This process converts the conservation equations into a system of algebraic equations, which is solved to obtain the dependent variables at the discrete element level.^171,172^ Discretization errors arise in both spatial and temporal domains, and the selected numerical method must ensure numerical stability and proper error conditioning throughout the simulation process. The numerical method selected must be able to maintain the error stable or well-conditioned during the simulation process.^173,174^

The finite volume technique is simple to implement several boundary conditions in a noninvasive way, which constitutes a great advantage of this method.^175^ Moreover, unknown variables can be evaluated at the centroids or in the vertex mesh cells. On the other hand, the FD method evaluates the solution at the boundary grid points, i.e., at the limits of the faces.^176^ Although the FD method is older than the FV method, it is gaining notoriety in CFD applications such as moving boundary problems, where boundary conditions must be updated during the calculation steps.^177,178^ Adaptive meshes can also be found in the MFEM. These meshes are time-dependent and can capture sharp moving fronts of the multicomponent adsorption process.^179^

The genesis of CFD modeling considers a transport equation in a general form for the adsorbate, considering homogeneous chemical reaction (eq 7), from which all conservation equations are derived (Andersson B., Andersson R., Hakansson L., Mortensen M., Sudiyo R. 2011; Wah-Yen et al. 2017):
15

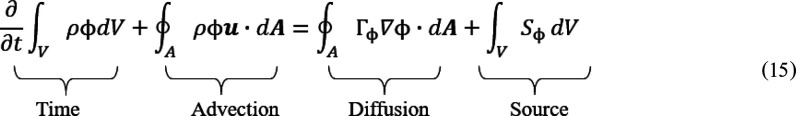

where ρ is the density, ϕ is a general scalar dependent variable, ***u*** is the velocity vector, Γ is the diffusivity, and *S* is the specific source term for a given ϕ. After integrating the conservation equation, variables will be obtained at each control volume centroid after interpolation procedures.^182,183^

Because the hyperbolic coupled set of PDE of momentum and mass conservation cannot be solved by analytical solution, the numerical methods FV, FE, and FD are often used for resolving the system of equations, in which the FV method is the most recurrent as a robust and rigorous tool for the treatment of nonlinear equations.^180,184,185^ Interpolation schemes applied to the FV method are widely reported in the literature.^183,186–190^ In the FV method, PDEs are discretized directly by numerical integration of each control volume applying the Gauss Theorem.^181^ The conservation property is based on the assumption that the inlet flow in a given volume is equivalent to the outlet flow to the adjacent volume.^182^ On the other hand, the FD method approximates the differential equations into finite difference equations predominantly by the Taylor series (Table 5).^177,191^

**Table 5:** Numerical Approximation of First and Second-Order Schemes for Spatial Derivatives by the Finite Difference Method^a^

numerical approximation schemes	derivative	truncation error	equation
backward difference (first order)	$\frac{\partial f}{\partial x}$	O(h)	$\frac{f\left(x\right)-f\left(x-\Delta x\right)}{\Delta x}$
backward difference (second order)	$\frac{\partial f}{\partial x}$	O(h^2^)	$\frac{3f\left(x\right)-4f\left(x-\Delta x\right)+f\left(x-2\Delta x\right)}{2\Delta x}$
backward difference (first order)	$\frac{\partial^{2}f}{{\partial x}^{2}}$	O(h)	$\frac{f\left(x\right)-2f\left(x-\Delta x\right)+f\left(x-2\Delta x\right)}{{\Delta x}^{2}}$
backward difference (second order)	$\frac{\partial^{2}f}{{\partial x}^{2}}$	O(h^2^)	$\frac{2f\left(x\right)-5f\left(x-\Delta x\right)+4f\left(x-2\Delta x\right)-f\left(x-3\Delta x\right)}{{\Delta x}^{2}}$
forward difference (first order)	$\frac{\partial f}{\partial x}$	O(h)	$\frac{f\left(x+\Delta x\right)-f\left(x\right)}{\Delta x}$
forward difference (second order)	$\frac{\partial f}{\partial x}$	O(h^2^)	$\frac{-3f\left(x\right)+4f\left(x+\Delta x\right)-f\left(x+2\Delta x\right)}{2\Delta x}$
forward difference (first order)	$\frac{\partial^{2}f}{{\partial x}^{2}}$	O(h)	$\frac{f\left(x\right)-2f\left(x+\Delta x\right)+f\left(x+2\Delta x\right)}{{\Delta x}^{2}}$
forward difference (second order)	$\frac{\partial^{2}f}{{\partial x}^{2}}$	O(h^2^)	$\frac{2f\left(x\right)-5f\left(x+\Delta x\right)+4f\left(x+2\Delta x\right)-f\left(x+3\Delta x\right)}{{\Delta x}^{2}}$
centered difference (second order)	$\frac{\partial f}{\partial x}$	O(h^2^)	$\frac{f\left(x+\Delta x\right)-f\left(x+\Delta x\right)}{2\Delta x}$
centered difference (second order)	$\frac{\partial^{2}f}{{\partial x}^{2}}$	O(h^2^)	$\frac{f\left(x+\Delta x\right)-2f\left(x\right)+f\left(x-\Delta x\right)}{{\Delta x}^{2}}$

a The series expansion is truncated in the desired order O (h), and as the order increases, the associated truncation error becomes smaller^192^.

Another numerical approach that has gained prominence in recent decades is the Lattice Boltzmann method due to the analysis of complex geometries in directly parallel codes that consider simultaneous collision and streaming effects.^121,193,194^ The technique consists of a small set of discrete particles used to represent mass, momentum, and energy transport. The density distribution function *f*_*i*_(*r*,*t*) portrays the particle status with a discrete velocity *e*_*i*_ at the position and time (*r*,*t*). Subsequently, velocity values are defined by the movement of particles from a lattice node to an adjacent node for each time step Δ*t*.^87,124,195^ Through the discrete collision operator (Δ_*i*_), the functions *f*(*r*,*t*) are reallocated in mesh nodes at each time step.^124^ The Lattice Boltzmann equation proposed by Benzi et al. (1992)^196^ is represented by eq 8.

16 $$f_{i}\left(r+e_{i}\Delta t,t+\Delta t\right)=f_{i}\left(r,t\right)+\Delta_{i}\left(r,t\right)$$

The main numerical solution techniques used in mass transfer and fluid flow simulations are represented in a schematic diagram (Figure 7) that briefly describes the calculation procedure of the respective conservation laws.

**Figure 7:**
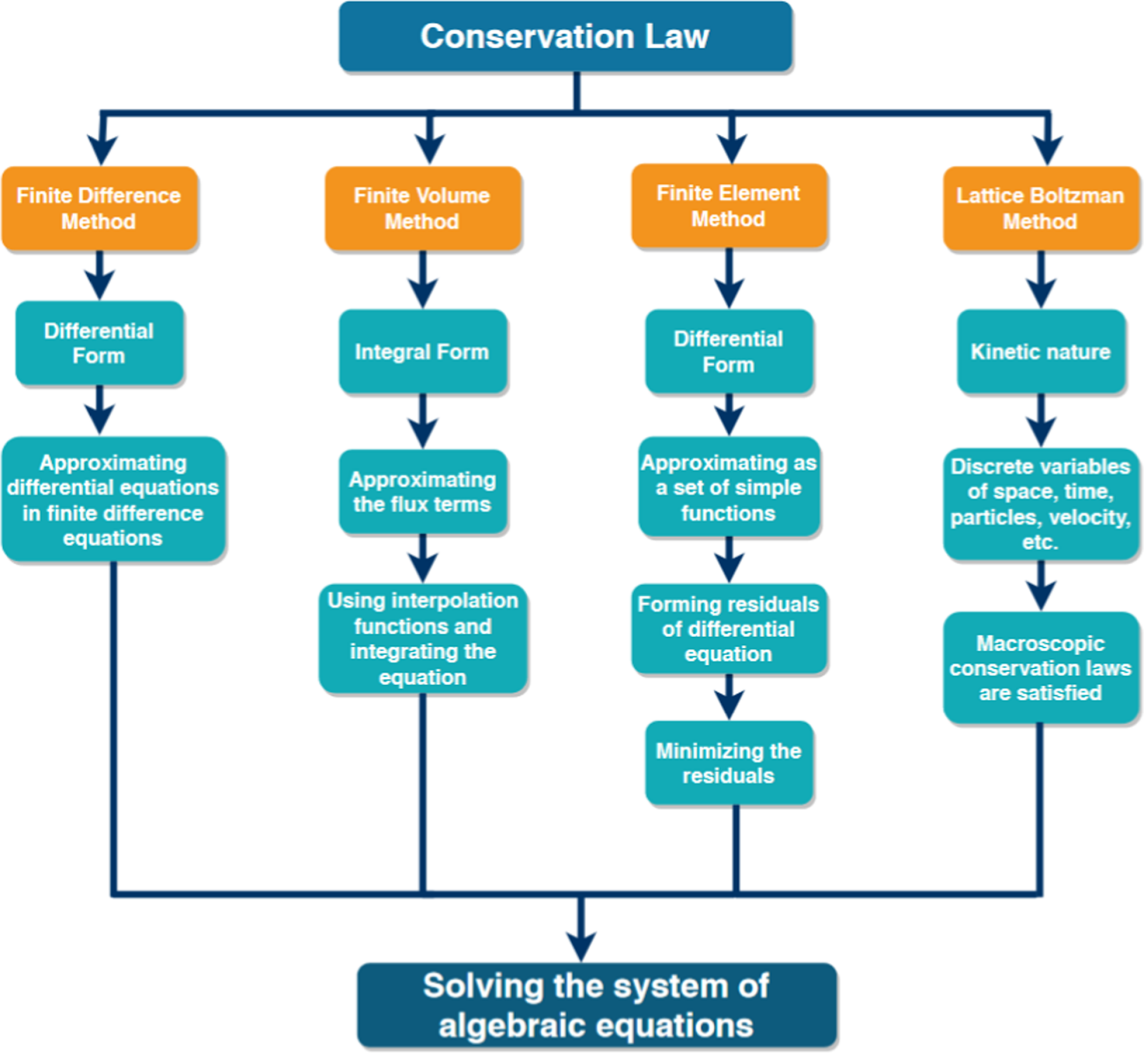
Comparative scheme of the steps involved in the application of the finite difference, finite element, finite volume, and Lattice Boltzmann methods.

Numerical consistency, robustness, convergence along numerical solutions, and computational cost should be carefully considered to define the most appropriate numerical method.

The finite volume approach is the most extensively used in terms of accuracy and performance for CFD applications. However, transient solutions are expensive due to the large set of PDEs required. In this case, the LB method presents a reduced computational cost for transient solutions. However, the LB method requires much finer meshes compared to the aforementioned methods. Therefore, the most appropriate numerical method for fluid dynamic analysis will depend on the purpose of the simulation and the computational resources available.

## 8 Conclusions and Perspectives

This work evaluated five elements of mechanistic modeling of monolithic chromatography: characteristics and geometric features of monolithic supports, models considering the precise structural properties, progress of solution methodologies, and verification procedures. There are few reported publications about chromatographic separation modeling applied to the monolithic stationary phases, especially those that integrate more geometric aspects, such as CFD models. However, in the recent decade, there has been a tremendous interest of the scientific community in this issue due to scientific discoveries, practical applications, and notably with the huge rise in computer capacity.

Mechanistic models should be able to simulate the mass transfer and momentum of monolithic matrices, replicating benchmark trials. However, their development faces hurdles linked to complex pore network morphology, scale concerns, and overparametrization. Furthermore, verification processes are typically overlooked, which generate gaps for model formulation. Scaling analysis could also be further studied as a consistent criterion for model simplification, offering intelligible descriptions of governing phenomena.

Future works should consider sensitivity analyses and apply CFD models to further improve the monolithic chromatographic system’s performance and stability. As a robust design tool, CFD models are essential for predicting, understanding, monitoring, and managing processes as well as generating quick and precise responses to scale and optimize such processes.

## 9 Future Trends in CFD Modeling: Practical Implications

The combination of experimental imaging techniques with machine-learning-based automated modeling has strong potential to improve the selection of the most relevant features in complex porous media. High-resolution imaging and 3D reconstruction enable detailed characterization of pore morphology; nevertheless, the wide variability in pore geometries and structural attributes—directly influencing diffusion, flow, and solute separation—makes the identification of meaningful features a persistent challenge. Although this discussion focuses on porous-media fluid dynamics, the proposed framework is broadly applicable to other domains that rely on structural characterization and predictive modeling.

Integrating artificial intelligence for computer-assisted engineering (AI-CAE) enables more robust and data-driven decisions. AI-based techniques enable automated calibration, uncertainty quantification, anomaly detection, and adaptive mesh or model refinement based on real-time data streams. AI models assist not only in selecting and optimizing multiple parameters but also in reducing dimensionality and computational cost by focusing on features with the greatest physical relevance. CFD models, in turn, facilitate rapid prototyping and provide deeper insight into the governing physical phenomena. The optimized features identified by AI can then be incorporated into predictive CFD simulations, resulting in a highly realistic representation of system behavior within a data-driven Digital Twin framework. Ultimately, this synergistic framework marks a paradigm shift from conventional offline simulations toward intelligent simulations, enabling real-time insight, predictive capability, and sustained process optimization.

## SYMBOLS

*a*: specific surface area (m^–1^)
*C*: adsorbate concentration in the bulk (kg/m^3^)
*C*_D_: adsorbate dimensionless concentration in the bulk
*C**: Adsorbate/adsorbent equilibrium concentration (kg/m^3^)
C_D_^*^: dimensionless equilibrium concentration of adsorbate/adsorbent
*C*o: Adsorbate concentration at the column inlet (kg/m^3^)
*D*: diameter of the cryogel column (cm)
*D*_AB_: molecular diffusion coefficient of the adsorbate in the fluid phase (m^2^/s)
*D*_ax_: Axial coefficient dispersion (m^2^/s)
d: Mean pore diameter (mm)
*d*_*i*_: Pore diameter of monolith i (m)
*d*_min_: minimum pore diameter (m)
g: gravitational acceleration (m^2^/s)
*k*_*w*_: hydraulic permeability (m^2^)
*k*_ad_: dimensionless adsorption coefficient
*k*_*f*_: mass transfer coefficient in the film (m/s)
*L*: length of the cryogel column (cm)
*L*s_i_: length of a streamline i (m)
*L*e_i_: distance between the ends of a streamline i (m)
*p*: static flow pressure (Pa)
Pe: Péclet number
*q*: Adsorbate concentration in the adsorbent (kg/m^3^)
*q*_*D*_: Dimensionless concentration of adsorbate in the adsorbent
*Q*_*V*_: Volume flow rate (m^3^/s)
Re: Reynolds number
Sc: Schmidt number
St: Stanton number
t: Flow time (s)
*t*_1_: Average residence time with a column bed (s)
*t*_0_: Average residence time without a column bed (s)
*u*: Superficial velocity of flow at each point of the domain (m/s)
*v*: Interstitial flow velocity at each point of the domain (m/s)
*V*_*V*_: Volume of the void space (m^3^)
*V*_*T*_: Total volume (m^3^)
*W*_1_: Specific gravity bottle weight filled with ethanol (g)
*W*_2_: Specific gravity bottle weight including ethanol and cryogel section (g)
*W*_3_: Specific gravity bottle weight measured after taking out ethanol-saturated cryogel section from *W*_2_(g)
*W*_*S*_: Ethanol-saturated cryogel section weight (g)
*z*: Column length coordinate (m)
*z*_*D*_: Dimensionless distance along the cryogel column
μ: Dynamic viscosity of adsorbate (Pa.s)
ρ: Density of adsorbate (kg/m^3^)
ρ_*e*_: Density of ethanol (g/cm^3^)
φ: Porosity of the cryogel column
τ: Tortuosity of the monoliths
τ_dmin_: tortuosity of a capillary with a diameter *d*_min_ (adimensional)
ω̅: fitting parameter given by slope of tortuosity vs capillary diameter (m^–1^)
γ: dimensionless empirical parameter
